# Rapid, repeatable landscape‐scale mapping of tree, hedgerow, and woodland habitats (THaW), using airborne LiDAR and spaceborne SAR data

**DOI:** 10.1002/ece3.10103

**Published:** 2023-05-25

**Authors:** David J. Luscombe, Naomi Gatis, Karen Anderson, Donna Carless, Richard E. Brazier

**Affiliations:** ^1^ Centre for Resilience in Environment, Water and Waste (CREWW) Faculty of Environment, Science and Economy University of Exeter Exeter UK; ^2^ Environment and Sustainability Institute University of Exeter Penryn UK

**Keywords:** change detection, land cover, LiDAR, SAR, trees, woodland

## Abstract

In the UK, tree, hedgerow, and woodland (THaW) habitats are key havens for biodiversity and support many related ecosystem services. The UK is entering a period of agricultural policy realignment with respect to natural capital and climate change, meaning that now is a critical time to evaluate the distribution, resilience, and dynamics of THaW habitats. The fine‐grained nature of habitats like hedgerows necessitates mapping of these features at relatively fine spatial resolution—and freely available public archives of airborne laser scanning (LiDAR) data at <2 m spatial resolution offer a means of doing so within UK settings. The high cost of LiDAR prohibits use for regular monitoring of THaW change, but space‐borne sensors such as Sentinel‐1 Synthetic Aperture Radar (SAR at ca. 10 m resolution) can potentially meet this need once baseline distributions are established. We address two aims in this manuscript—(1) to rapidly quantify THaW across UK landscapes using LiDAR data and (2) to monitor canopy change intra‐ and inter‐annually using SAR data. We show that workflows applied to airborne LiDAR data can deliver THaW baselines at 2 m resolution, with positional accuracy of >90%. It was also possible to combine LiDAR mapping data and Sentinel‐1 SAR data to rapidly track canopy change through time (i.e., every 3 months) using, cloud‐based processing via Google Earth Engine. The resultant toolkit is also provided as an open‐access web app. The results highlight that whilst nearly 90% of the tallest trees (above 15 m) are captured within the National Forest Inventory (NFI) database only 50% of THaW with a canopy height range of 3–15 m are recorded. Current estimates of tree distribution neglect these finer‐grained features (i.e., smaller or less contiguous THaW canopies), which we argue will account for a significant proportion of landscape THaW cover.

## Graphical abstract

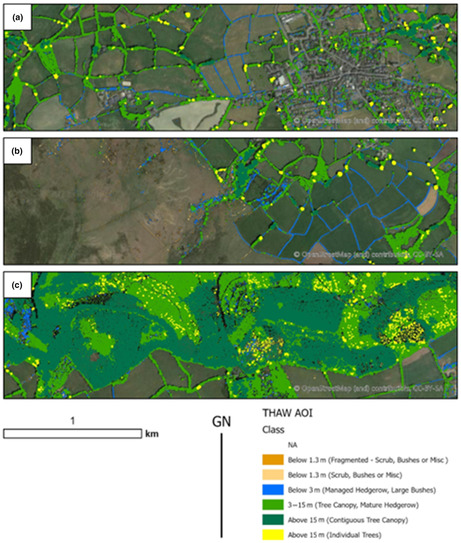

## 1 INTRODUCTION

Trees, Hedgerows, and Woodlands (THaW) are a major landcover component across temperate landscapes such as the UK, where broadleaf deciduous woodland forms the natural climax community. In the UK, the landscape is now dominated by arable and pastoral agriculture, with woodland habitats (stands of trees with a canopy cover of at least 20%) persisting as fragments of varying size, and as linear analogs of woodlands which double as enclosure boundaries (hedgerows) and wildlife corridors (Axe et al., 2017; Staley et al., 2012). At the national scale THaW habitats support a huge diversity of native or naturalized flora and fauna (e.g., oak‐dominated woodland supports ca. 2300 species in the UK; Mitchell et al., 2019). UK treescapes are also estimated to hold 584 Mt CO_2_ in woodland standing biomass (Morison et al., 2012). Despite this considerable biodiversity and carbon stock value, the UK is one of the least densely forested countries in Europe (at 13%) (https://www.forestresearch.gov.uk/; Langsdorf et al., 2022) yet with a strong potential for expansion with appropriate management (Morison et al., 2012) and increasing political desire to do so (e.g., England Trees Action Plan 2021–2024 – GOV.UK, n.d.).

Whist THaW in the UK are protected under the Wildlife and Countryside Act, 1981 and The Conservation of Habitats and Species Regulations, 2017 they are also under increased threat and pressure from development, intensive agriculture, and poor or inappropriate management (Staley, Amy, et al., 2012; Staley, Sparks, et al., 2012). For example, hedgerows have suffered extensive loss during the 19th century (Staley et al., 2012) with up to 50% being removed (Petit et al., 2003) to improve agricultural intensification and mechanization. Similarly, many remaining hedgerows are cut (flailed) or managed annually, limiting the average height to less than 2 m (Axe et al., 2017) which is substantially shorter than would be expected naturally most UK hedgerows comprise a mix of trees such as beech (*Fagus sylvatica*), sycamore (*Acer pseudoplatanus*), elder (*Sambucus nigra*), hawthorn (*Crataegus monogyna*), and woody shrubs such as blackthorn (*Prunus spinosa*) or gorse (*Ulex europaeus*).

Understanding the patterning and distribution of these habitats is critical to identifying opportunities for habitat expansion, creation, carbon sequestration, ecological restoration, and management (Bateman et al., 2022). As countries such as the UK consider how to achieve the objective of net zero by 2050 and undergo political realignment of agricultural policy, with respect to natural capital and climate change adaptation and mitigation (e.g., Environment and land management scheme, ELMS; 25 Year Environment Plan–GOV.UK, n.d.), it is now critical to be able to routinely quantify THaW habitats, their spatial distribution, and dynamics. Trees outside of woodlands, including most hedgerows, are of specific concern in this respect, given that the many Land Use and Land Use Change (LULUC) datasets only describe woodland that exceeds an area threshold (Morton et al., 2011)—for example, the UK's National Forest Inventory only includes woodland measuring greater than 0.5 hectares in area, with a minimum width of 20 m, and greater than 20% tree canopy cover (Smith et al., 2010).

**FIGURE 1:**
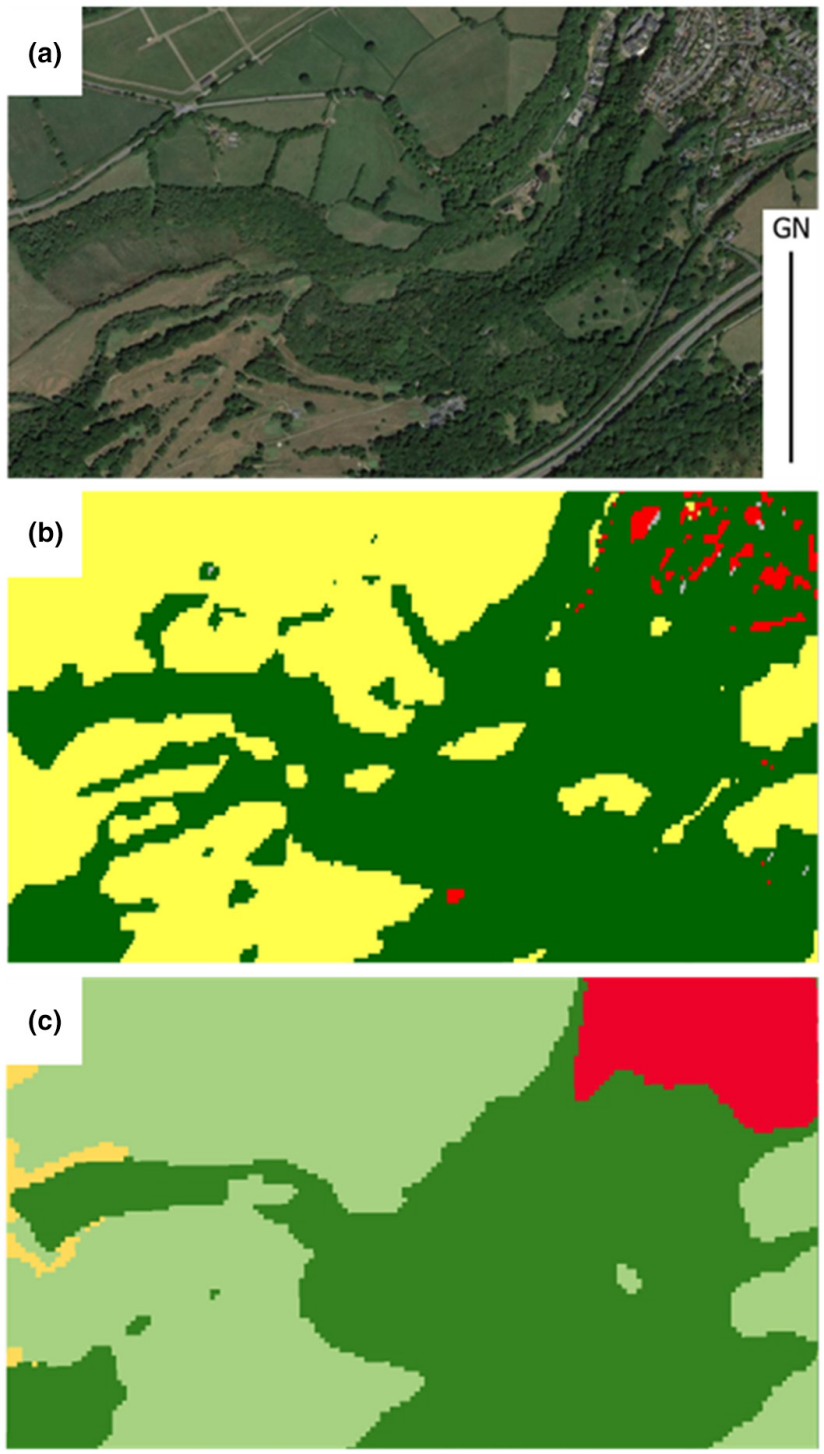
Example of (a) aerial photography (©Getmapping Plc) of mixed Tree Hedgerow and Woodland (THaW) cover across an urban–rural transition within the North Devon Biosphere Reserve. (b) Land cover map using ESA WorldCover 10 m 2020 mapping and (c) ESRI Global LULC 10 m mapping, across the matching extent. For both b and c dark green areas represent THaW cover, but it is evident that narrow features such as hedgerows around field boundaries and small stands of trees or individual trees are omitted by both products.

Surveying of THaW habitats usually relies on a combination of manual field surveys, manual digitization of aerial photographs, or classification of optical remote sensing datasets captured from airborne or spaceborne platforms, to generate land cover mapping products (Lira et al., 2012; Figure 1). Both manual field survey and manual digitization are laborious (and thus expensive), which limits the spatial coverage of such baselines. Previous work has shown that satellite‐based approaches for mapping THaW distribution in heterogeneous systems such as urban environments deliver biased (usually underestimates) of tree cover because trees tend to occur in distinct patches at grains finer than the ground sampling distance of a single pixel (e.g., 10 m for Sentinel 2 data (Casalegno et al., 2017)).

Figure 1 provides an example, showing how national/international scale remote sensing classification approaches provide some useful data for mapping large contiguous woodland stands, but miss the detail of small stands of trees or narrow features, such as hedgerows, owing to a mismatch between the relatively coarse spatial resolution of the data compared to the grain of the THaW features (Brown & Fisher, 2012). Areas mapped as urban in both the ESA (Figure 1b) and ESRI LULIC (Figure 1c) mapping products are colored red. These maps highlight the problems of mapping THaW cover in such complex areas of landcover. Similarly, the dark green features in both images are the extent of mapped THaW cover, illustrating the lack of spatial detail, and mapping of trees outside of woodland.

Unlike automated land use mapping (above), other studies have used remote sensing data to derive tree mapping products using a combination of aerial photography, color infrared imagery, and airborne LiDAR, across landscape extents (Eddy James, 2022). Whilst providing robust data, such methods are only available commercially, are truncated to trees >3 m, and rely on episodic revision for any useful change detection metrics. Over defined extents, it is even possible to use LiDAR data to map and characterize individual tree crowns, by segmenting individual crown morphologies to a larger extent (Aubry‐Kientz et al., 2019; Mongus & Žalik, 2015; Yun et al., 2021). Further studies have mapped the extent and distribution of woody linear features in the UK using ancillary mapping products, which describe the field boundaries and vegetation height attributes, although this only covers part of the landscape THaW cover and provides outputs only as vector line data (Scholefield et al., 2016). Approaches using multiband SAR and LiDAR data to quantify THaW habitats across site‐specific extents are particularly useful in understanding the three‐dimensional vegetation structure and how this relates to regional biodiversity and habitat management (Bergen et al., 2009). However, these have been less appropriate for landscape or regional scale upscaling, due to the methods used, including SAR interferometry (Balzter et al., 2007) which is computationally expensive. Where such sensors have been applied, they have been found to provide robust measurements of habitat extent, change, and threats to these structurally complex habitats including forests and trees (Nagendra et al., 2013). These examples, alongside numerous site‐specific uses of LiDAR for woodland mapping (Lefsky et al., 2002; Streutker & Glenn, 2006; Vierling et al., 2008; Zimble et al., 2003) illustrate the unmet potential for a unified THaW mapping product that can deliver key outputs in an open access format.

Recent improvements in remote sensing technologies, data availability (McGarragh et al., 2015), and cloud data processing methods (e.g. Google Earth Engine; Gorelick et al., 2017) have the potential to facilitate a step change in the ability to describe the distribution and monitor the dynamics of THaW habitats. In combination, these technologies now provide a means to derive landscape‐wide, physically based measurements of THaW cover and change, across national extents and in unparalleled spatial (e.g., LiDAR data with a spatial granularity of <2 m^2^) and temporal (e.g., Sentinel 1 SAR with sub weekly return periods) detail. There is thus further potential to combine THaW products derived from remote sensing analyses with modeling to estimate biomass and carbon stocks (building on the work of Bateman & Lovett, 2000).

The aims of this study were to:

1. Measure, map, and quantify THaW baselines across UK landscapes using freely available LiDAR data.
2. Develop a rapid cloud‐based workflow to determine canopy cover change compared to the product from (1) across intra‐ and inter‐annual time steps, using freely available Sentinel‐1 SAR data.

THaW maps are needed to answer questions that relate to public‐facing agendas around carbon, biodiversity, and environmental management. To ensure that the results could be used for the public good, a third aim was therefore:

3 To deliver a web‐based tool, to unlock such information from open‐source data assets for public audiences.

## 2 METHODS

### 2.1 Study location

The chosen study landscape encompassed the North Devon UNESCO Biosphere Reserve and Dartmoor National Park both located in the southwest of the UK. These areas form a contiguous landscape block covering more than 3800 km^2^. This extent has an elevation range between sea level and 621 meters and demonstrates a diversity of landscape character types and habitats including urban, intensively managed farmland, commercial forestry, primary travel corridors, and open upland moorland with blanket bog habitats (Gatis et al., 2022). As such, this area provides a sufficient breadth of land cover types to test the robustness of the mapped THaW habitats and the detection of canopy change.

### 2.2 LiDAR data

Open‐source airborne LiDAR Digital Surface (DSM) and Digital Terrain Model (DTM) products from the NERC Tellus project were selected. Data were collected in the summer and autumn 2013 (Ferraccioli, 2014). 2 m DSM and DTM products were downloaded from catalogue.ceh.ac.uk and processed in Arc GIS Pro Version 2.3.2 (ESRI Inc).

### 2.3 SAR data

Sentinel 1 SAR polarimetric data were acquired and automatically pre‐processed in Google Earth Engine (https://earthengine.google.com) using the ESA Sentinel‐1 Toolbox. Both the thermal noise removal and radiometric calibration tools were used to pre‐process the data. Data were also terrain‐corrected using SRTM 30. The resultant terrain‐corrected values used are in decibels via log scaling conversion. Multiple 3‐month temporal aggregations of the S1 Grid product were computed (Canty et al., 2019; Mullissa et al., 2021; Quegan & Jiong Yu, 2001) using interferometric wide swath instrument mode and vertical to horizontal (VH) transmitter/receiver polarization to maximize the sensitivity of the instrument to canopy volume scattering.

### 2.4 THaW baseline model

**FIGURE 2:**
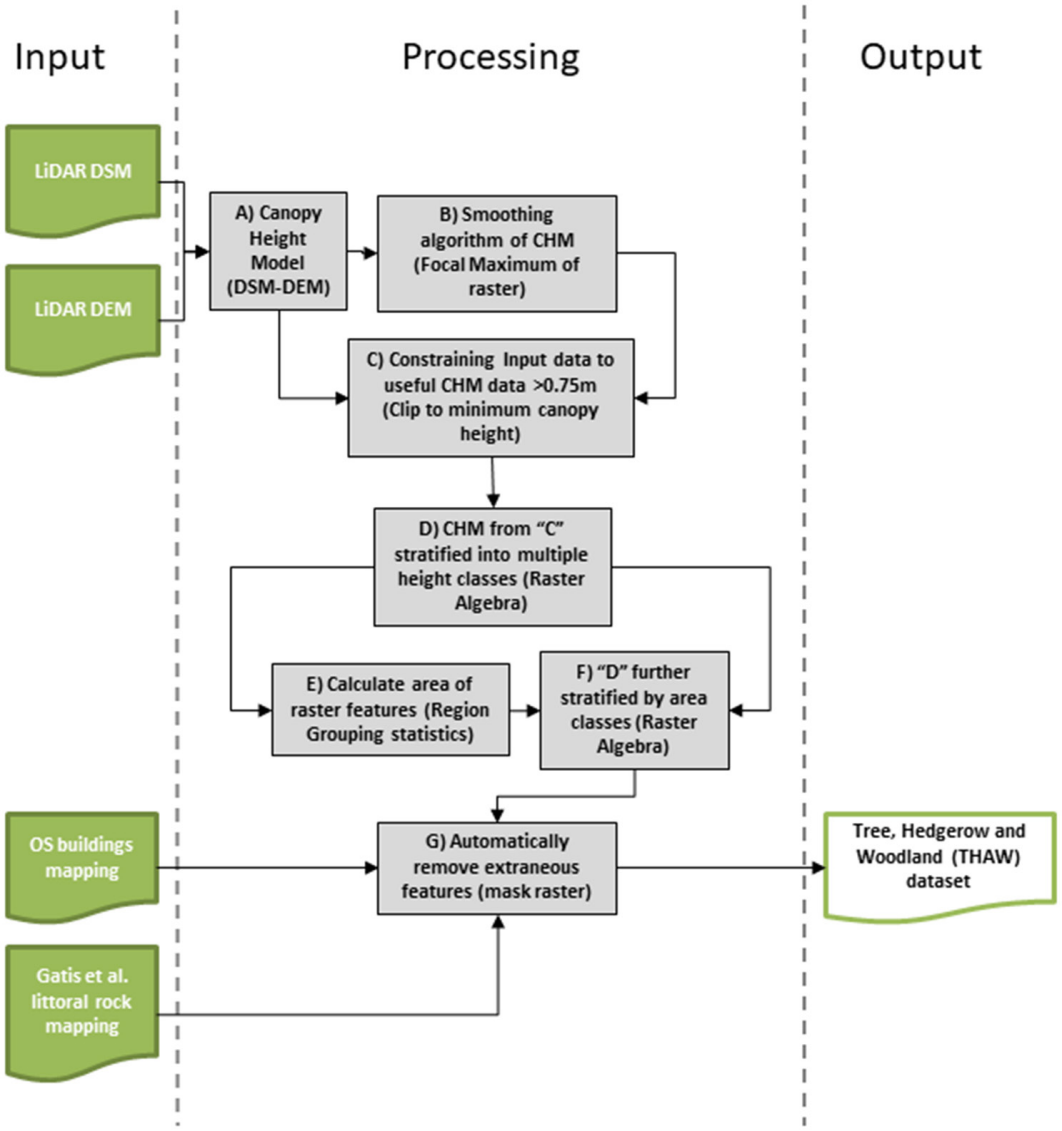
Tree Hedgerow and Woodland (THaW) Baseline Model data processing pipeline. Summarizing the method by which the THaW features were automatically extracted from the input LiDAR data.

The THaW workflow is outlined in Figure 2. THaW features were automatically extracted from the input LiDAR data using raster classes based on both their planar and vertical geometry (Table 1). These geometries were based on an empirical canopy height model (CHM) calculated from the LiDAR Digital Surface (DSM, first return) and Digital Terrain Models (DTM, last return). These were then spatially filtered to simplified mapped THaW habitats, as below.

**TABLE 1:** User‐defined Tree Hedgerow and Woodland (THaW) classes were applied in the processing pipeline (Figure 2).

Grid code	THaW class description
0	Null/Non THaW habitats, Below 0.75 m (Scrub, Bushes, or Misc)
5	0.75–1.3 m (Fragmented—Scrub, Bushes, or Misc, e.g., fence/dry stone wall <10 m^2^)
1	0.75–1.3 m (Scrub, Bushes, or Misc >10 m^2^)
2	1.3–3 m (Managed Hedgerow, Large Bushes)
3	3–15 m (Tree Canopy, Mature Hedgerow)
4	Above 15 m (Contiguous Tree Canopy)
6	Above 15 m (Emergent or isolated trees)

The data processing pipeline illustrated in Figure 2 initially separated the CHM into height‐based classes, based on a 6 by 6 m moving window of the local maximum CHM, the output from this was constrained to heights above 0.75 m. Next, standard region grouping methods were used to compute the feature area of every individually mapped canopy component to enable area‐based categorization. This stage was implemented through the ArcGIS spatial analyst extension using the region grouping tool and conditional raster calculations. The calculated area of all features was then used to further categorize and encode THaW habitats based on the classes in Table 1. The height/area classes were decided in collaboration with land managers and representatives from the UK forestry commission, based on assumptive definitions of canopy types. In combination these are designed to discriminate THaW vertically, (i.e., hedgerows from maturing trees) and by area, that is, scrub from hedgerow, or veteran trees from larger stands of mature trees (>15 m). This latter class is of specific importance for monitoring the loss of protected trees in the UK landscape.

Finally, features known to result in false positives (via iterative examination of trial output data) were removed as a raster mask using other spatial land cover datasets, including mapping of buildings and scree/outcrop rock formations (Gatis et al., 2022). These features were buffered and co‐processed to autonomously remove these false positives from the finalized dataset (Figure 2‐G). The THaW data pipeline is an entirely raster‐based approach, mitigating any requirement to polygonize the >5 million data objects (in the study area of 3800 km^2^) at any intermediate stage. This approach, combined with the parallelization of computation tasks significantly increases the processing speed of the model, as well as performance stability. Computation of the THaW dataset for the entire study area using this method was completed in <30 min using a relatively high‐specification desktop workstation (i7‐8700K CPU, 64.0 GB DDR3 RAM, NVIDIA Quadro P400 GPU). This protocol was also built into an Arc Pro compatible toolbox to enable the application of this method across other extents with available LiDAR raster data.

### 2.5 THaW change detection

Sentinel‐1 (S1) SAR data were used to provide an index of change in land surface structure. The data used were pre‐processed using ESA Sentinel‐1 Toolbox, in line with the methods outlined in the Earth Engine Data Catalogue, to minimize the effect of terrain and topographic distortions. Individual overpasses of these data provide a visualization of radiometric backscatter that can be useful in describing canopy volume structure and complexity over multiple periods. The resultant dataset is, however, subject to “salt and pepper” type noise (Singh et al., 2021), due to atmospheric and sensor inconsistencies causing uncertainty in the extent of observable structures. Here, we use temporal composites to improve the long‐term representation of land surface structure in the dataset, without using any low‐pass filtering that may have eroded data sensitivity (Mullissa et al., 2021; Singh et al., 2021). All calculations were undertaken in Google Earth Engine (GEE) enabling the selection of the relevant temporal window by the user, via a bespoke graphical user's interface. The SAR data used to derive the composites were taken from the Copernicus Sentinel 1 ground range detected (GRD) image collection available from GEE. The instrument mode was set as informetric wide swath (IW) with descending orbit passes (to minimize changes in look angle) and vertical to horizontal (VH) transmitter/receiver polarization. VH polarization was used to increase the detection of backscatter due to canopy volume scattering.

**FIGURE 3:**
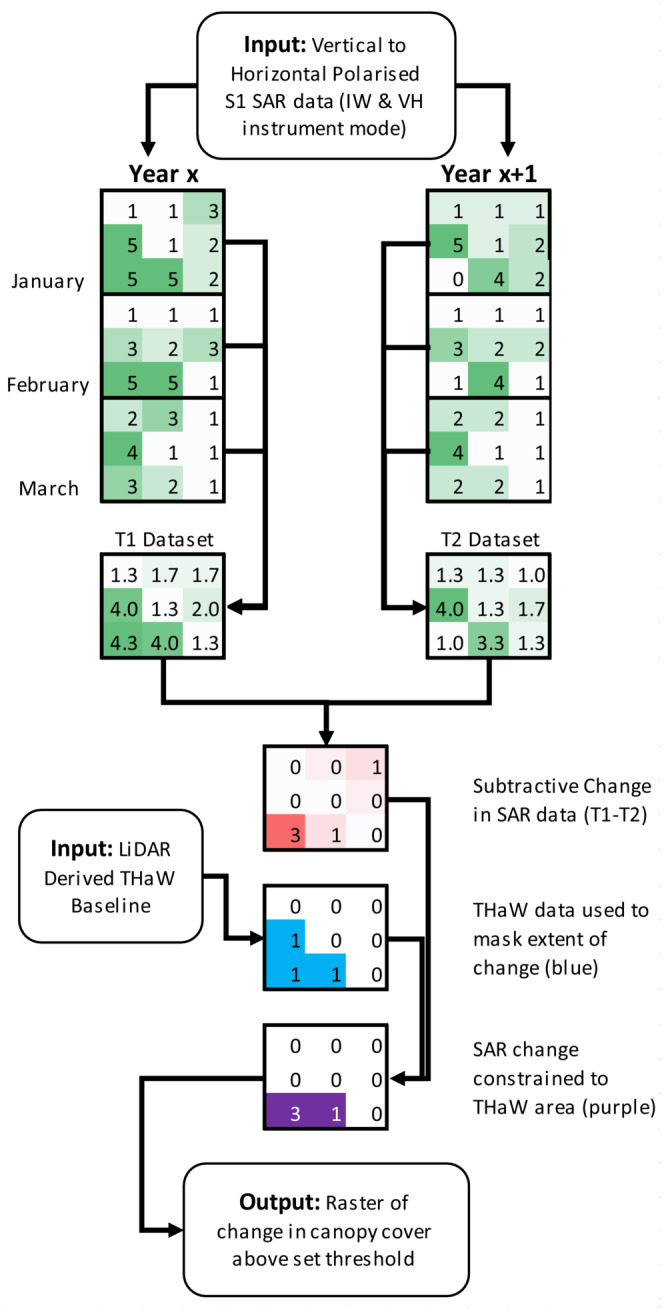
Conceptual schematic of Synthetic Aperture Radar (SAR) Sentinel‐1 based canopy change detection constrained by the Tree, Hedgerow, and Woodland (THaW) dataset. Here, a conceptual 3 × 3 raster illustrates how temporal composites of SAR data (green) are used to detect change (red). This is then constrained by the mapping illustrated in Figure 2 (blue) to derive a discreate map of change detection (purple).

As illustrated in Figure 3, the temporal composites were used to increase the signal‐to‐noise ratio of surface structures and therefore reduce the likelihood of false positive canopy change artifacts in the resultant data. The multitemporal composites output a raster of the mean average backscatter value (in dB) for each pixel from all observations (ca.18) during the three‐month window. While this process can reduce the detection of change attributable to disturbance within the observation window, the examination of sequential temporal composites minimizes the loss of information.

Figure 3 illustrates the change detection data processing employed as a conceptual 3 × 3 raster. Change is detected by comparing canopy volume scattering, as measured by the S1 GRD products, for any selected calendar quarter (Year = x + 1) to a matching time window from the previous year (Year = x). Resultant change data (red) were then truncated to between 1 and 3 dB and constrained to the spatial extent of the THaW baseline dataset (blue) within GEE to remove the contribution of wider landcover change in the detected features. For example, fluctuating crop cover, changes in urban structure, changes in surface water, and earthworks. This method also limits the confounding effects of intra‐annual variability in atmospheric conditions, ground conditions, and canopy leaf habit, by comparing matching calendar quarters in the preceding year, thereby minimizing seasonal differences in surface moisture content and structure.

## 3 RESULTS

### 3.1 Aim 1: Measure, map, and quantify THaW baselines across UK landscapes using freely available LiDAR data

**FIGURE 4:**
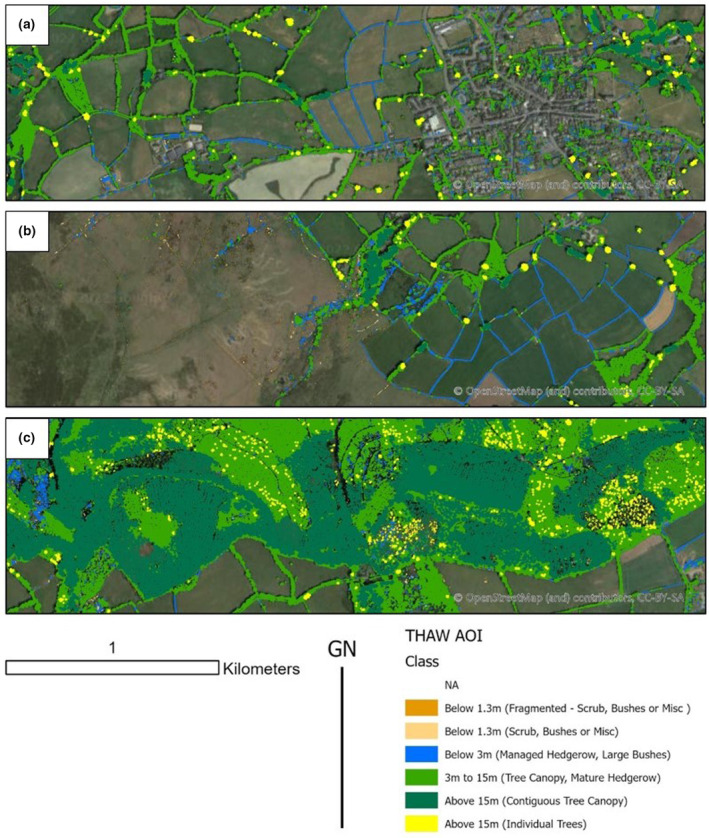
Tree Hedgerow and Woodland (THaW) mapping data across contrasting landscape areas within the study area. (a) transitional woodland to urban canopy cover. (b) farmland to moorland transition. (c) Contiguous mixed woodland cover.

The baseline THaW model provides a dataset describing the position and extent of tree, hedgerow, and woodland (THaW) classes, as illustrated in Figure 4 and the associated web tool (see section 7). The thresholds selected to separate and delineated contiguous tree canopies are shown to define forest compartments effectively within both conifer plantation blocks (Figure 4c) and broadleaf plantations. Figure 4b illustrates a scrub‐to‐woodland transition across an upland moorland fringe, which is also visible in the underlying aerial photography. Hedgerows are mapped as a separate geometry class. This effectively delineates the hedgerow network, including gate gaps and positions where features separate (Figure 4a,b). In addition, these data illustrate where hedgerows are degraded and/or fragmented, sometimes into “scrub” class features. The position and extent of urban trees and other woody structures such as garden hedges and larger bushes are also mapped effectively (Figure 4a) when using appropriate ancillary data to autonomously remove buildings.

As well as describing the spatial distribution of these groups of THaW structures, the data also illustrate the network connectivity of these mapped features and may be readily used for habitat connectivity analysis. Given these products are deterministically generated, they were also subject to some false positive classifications, although these were relatively uncommon. For example, solar farms were mapped as hedgerow features; parked cars were mapped as bushes, and dry stone walls were mapped as a scrub (these can be seen in the web tool—see Section 7).

Table 2 provides accuracy metrics for the mapped THaW areas within three areas of interest covering differing canopy types (Figure 4). Within these areas, THaW mapping was, compared to manually digitized THaW features from open‐source aerial photography of a similar age. Using a confusion matrix approach, the overall THaW mapping data are shown to perform well and consistently across the landscape extent. Accuracy varies between 94% for managed hedgerows (Kappa Coefficient 0.97), 93% for woodland areas (Kappa Coefficient 0.84), and 91% for mature hedgerow canopies (Kappa Coefficient 0.94). User's accuracy for THaW features is shown to be more variable, ranging from 63% to 98%. These data go some way to assess the positional accuracy of the THaW mapping outputs, but due to the parallax and georectification uncertainty associated with true color aerial photography, digitized data products are not inherently more accurate than the LiDAR‐derived THaW data, and the opposite may also be true.

**TABLE 2:** Comparison of Tree Hedgerow and Woodland (THaW) mapping data to manually digitized features based on Google Earth Pro 2017, across three areas of interest covering three key canopy types.

	Mature hedgerow	Managed hedgerow	Woodland block
A – Area in THaW m^2^	16,186	4443	85,822
B – Area digitized m^2^	19,421	3622	91,523
% Agreement of total mapped area A/B	83%	123%	94%
User's accuracy tree	91%	63%	98%
User's accuracy not tree	91%	98%	77%
**Overall accuracy**	**91%**	**94%**	**93%**
**Kappa coefficient**	**0.94**	**0.97**	**0.84**

*Note*: Bold values indicate the significance of the summary stats.

**FIGURE 5:**
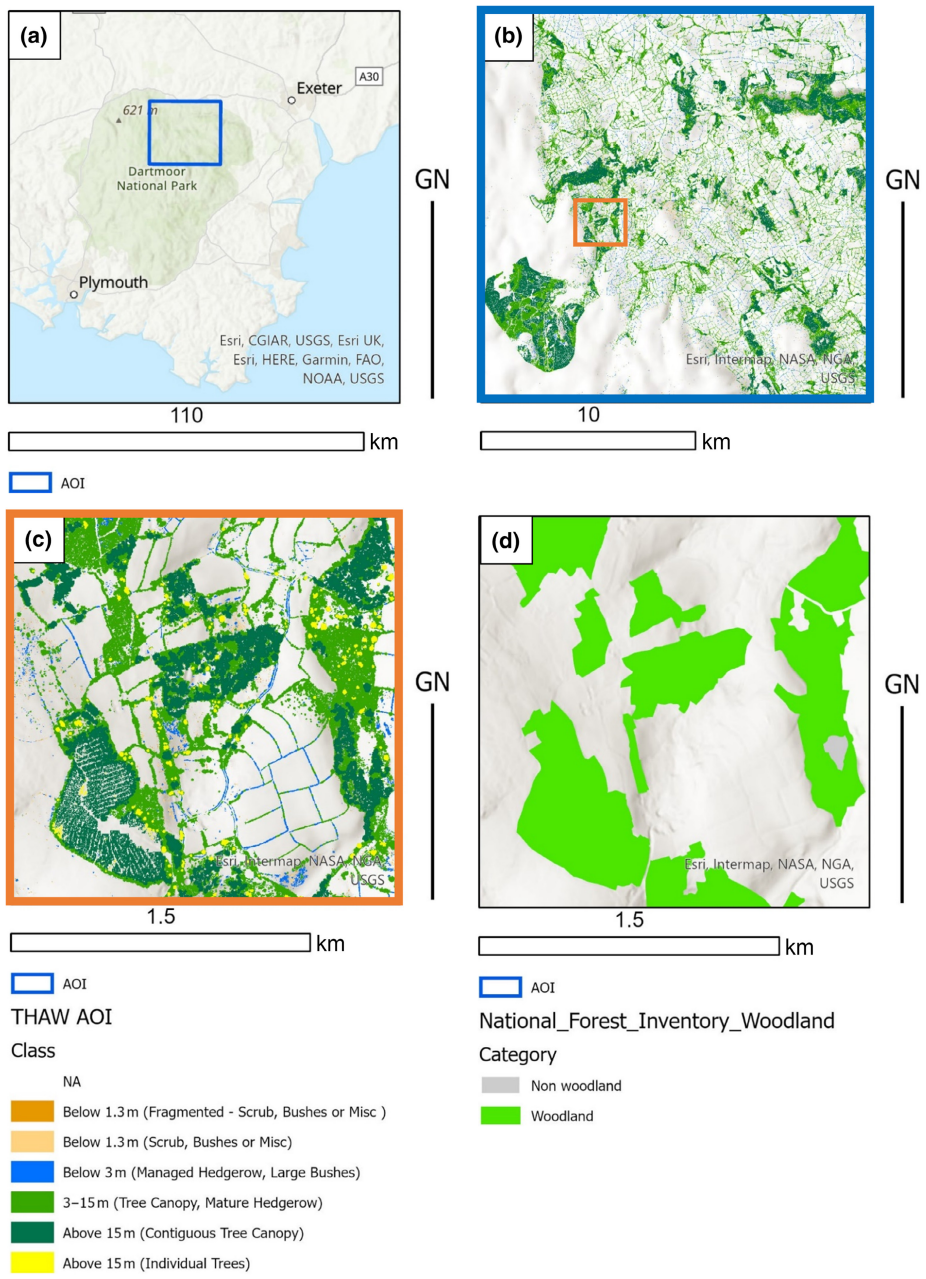
(a) Position of focus area in SW England, (b) THaW mapping data across the focus area. (c) close‐up of THaW mapping data from the orange square in b. THaW mapping derived from the process outlined in Figure 2. (d) National Forest inventory data (Smith et al., 2010) provided by the Forestry Commission England for the same extent as c.

Figure 5 illustrates the greater detail and canopy separation achieved by the THaW mapping when compared to the UK National Forest Inventory (NFI) Produced by the UK Forestry Commission. Unlike NFI the THaW data also contains information on the relative height of the canopy components and demonstrates a greater total extent of mapped THaW cover. This is expected given the spatial resolution and designed use of the NFI data but illustrates that trees outside of woodland are a significant component of canopy cover in the UK. The THaW mapping data trace connectivity between NFI woodland compartments and enables detailed, quantitative investigation of habitat structure, including in volumetric dimensions.

**FIGURE 6:**
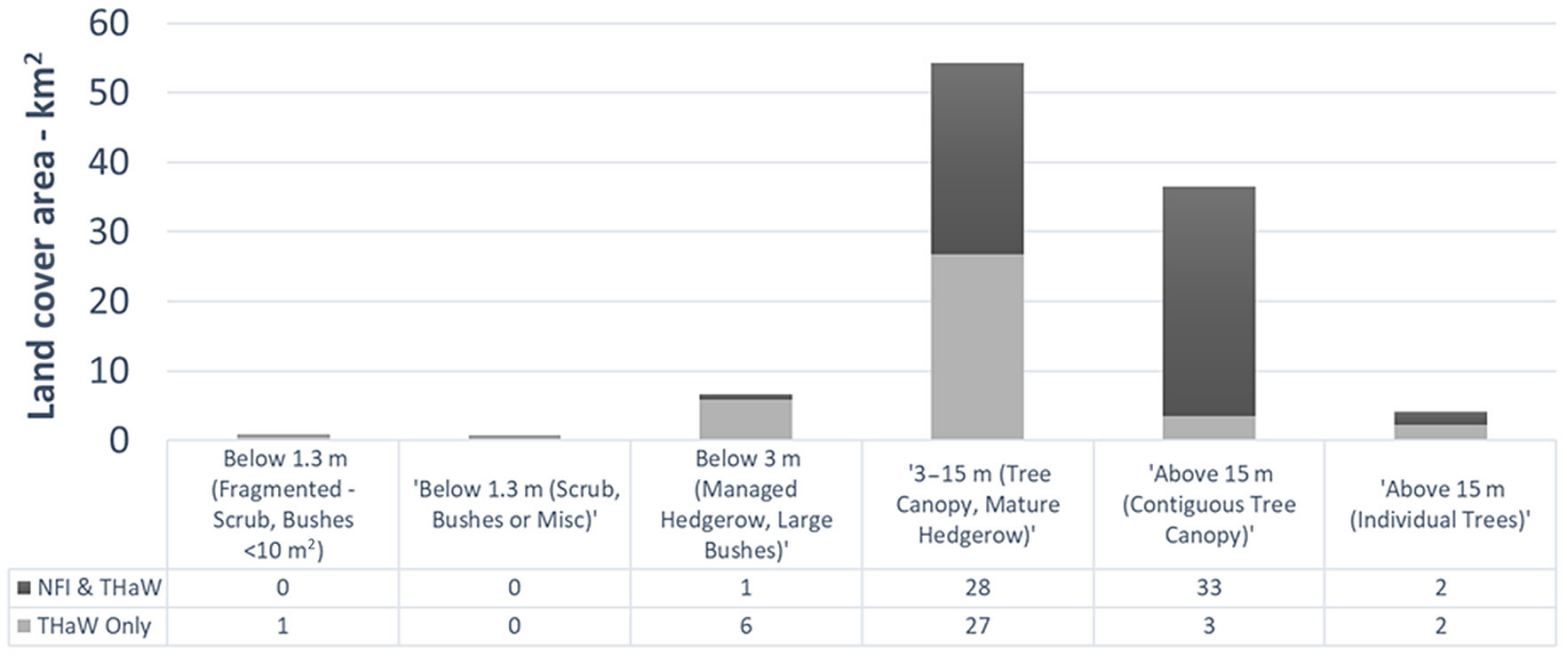
Area of THaW cover (km^2^) that is also captured by the NFI dataset (Dark) or only captured in THaW (Light).

Whilst nearly all (90%) trees above 15 m are captured within the NFI dataset (Figure 6), only 50% of the canopy cover from 3 to 15 m is included in NFI. This illustrates the significant contribution that shorter trees outside of woodland make to the canopy cover of the UK and how they are underrepresented in NFI. Additionally, almost all hedgerow is excluded from the NFI dataset, as would be expected, given its minimum mappable unit (Smith et al., 2010).

### 3.2 Aim 2: Monitor canopy change across intra‐ and inter‐annual time steps using freely available Sentinel‐1 SAR data

**FIGURE 7:**
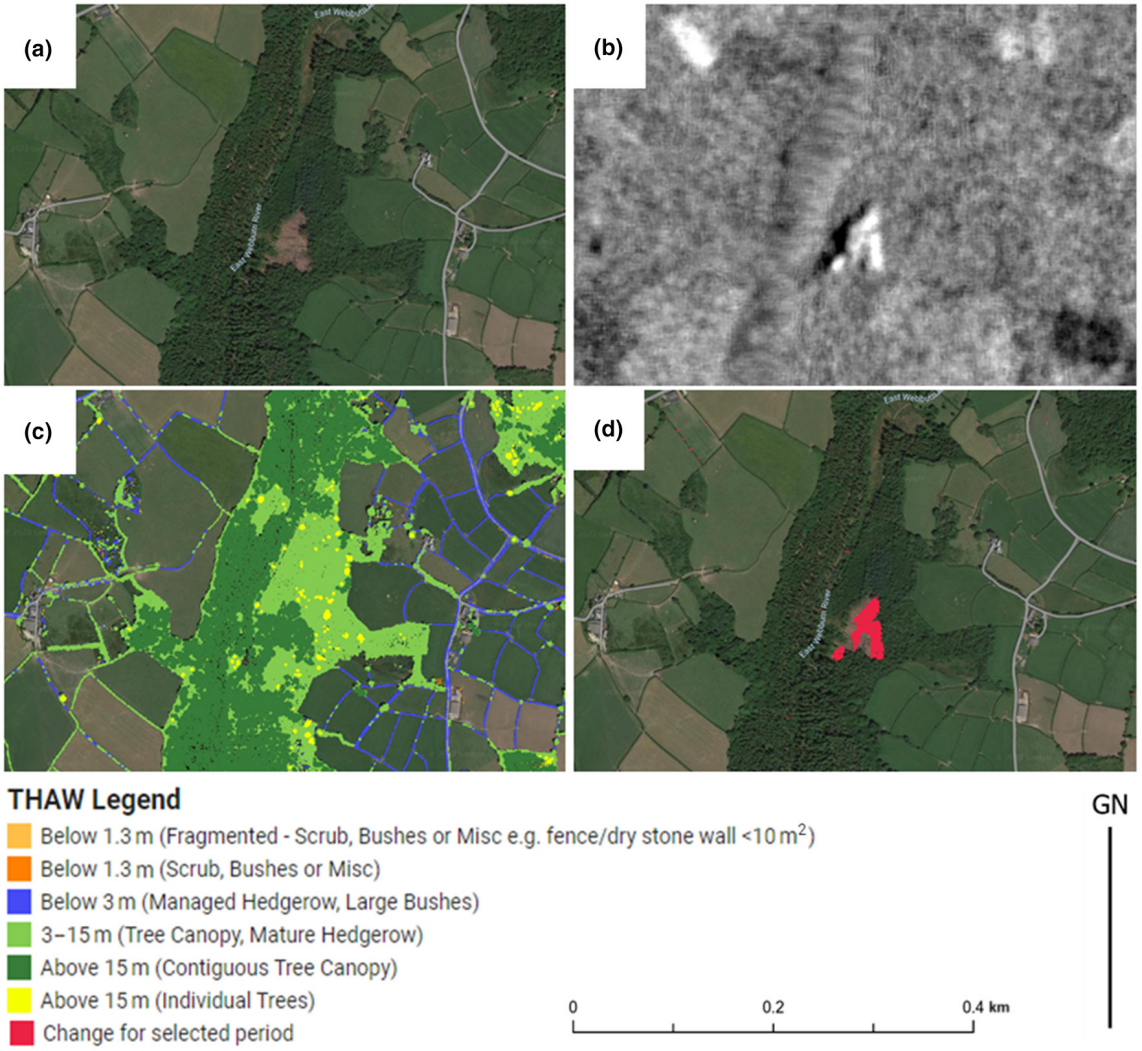
(a) Aerial image (© Getmapping Plc) showing an area of clear‐fell. (b) difference in SAR backscatter (dB) between a timeseries of quarter 1, 2020 to quarter 1, 2019, (c) output Trees, Hedgerows, and Woodlands (THaW) mapping data, (d) extracted extent of change derived from THaW mapping and SAR data.

When examining the change detection dataset using the web tool (see section 7), certain time periods (for example Quarter 42,019) produce numerous defined areas of canopy loss across a range of THaW types (for example, Figure 7d). However, some other time periods (e.g., Quarter 2, 2022) suffer more from small extents of assumed false positive artifacts or “noise”. Figure 7 illustrates the extraction of a constrained extent of canopy change (due to clear felling) using SAR and THaW data. The example given demonstrates that the method used is able to effectively describe defined extents of loss (e.g., Figure 7d) resulting from the S1 SAR data processed to indicate surface structure change/loss (Figure 7b) having a threshold applied and then constrained by the THaW mapping data from LiDAR (Figure 7c). The results indicate that the spatial extent of canopy loss can be quantified using this method.

**FIGURE 8:**
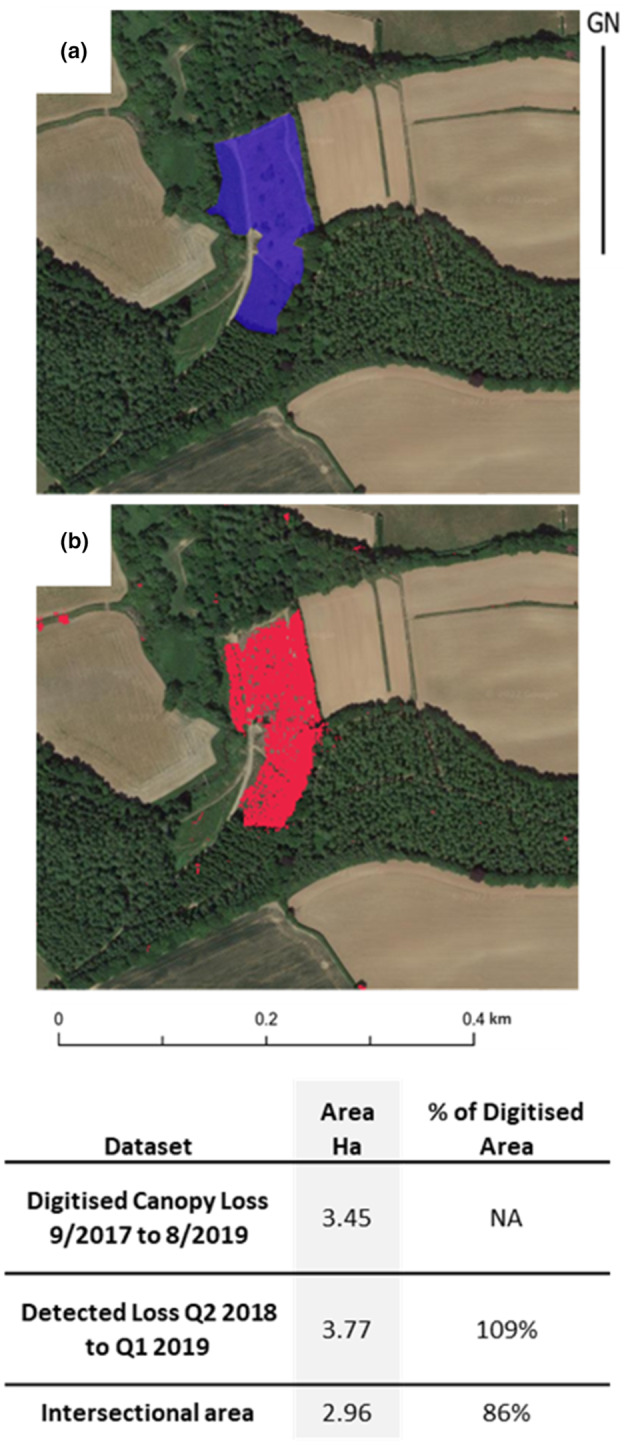
Comparison of (a) digitized extent of clear‐felling, between 2017 and 2018, with (b) the output from the change detection methodology which occurs on quarter 2 (April–June) 2018. Aerial imagery (© Getmapping Plc).

The result of this analysis provides numerous extents of detected change across the entire study area, for any given change detection period selected. These include change detection within woodland blocks, in hedgerows, and isolated tree canopies. Recent underlying aerial photography available as a default layer in Goggle Earth Engine, often confirms the detection of significant clear‐felling. To aid in understanding the positional accuracy of these extents, we have compared a digitized extent of clear‐felling, from a known time window, with the output from the change detection methodology (Figure 8). These data illustrate good correspondence between the two extents, with less than 10% deviation in the total measured area. Such discrepancies may be expected given the granularity of the SAR data used (10 m) and the uncertainty inherent with manual digitization of aerial photography.

This example (Figure 8) also illustrates how using quarterly composites of SAR data enables the window in which the felling occurred to be better constrained (April–June 2018) than aerial imagery (September 2007–August 2019). The digitized extent of woodland removal, shown in blue is compared to the automatically mapped extent shown in red.

## 4 DISCUSSION

### 4.1 Aim 1: Measure, map, and quantify THaW baselines across UK landscapes using freely available LiDAR data

The need to measure, map, and quantify THaW across UK landscapes is imperative in the context of shifting land use management, land use change, and forestry policy. The simple deterministic modeling/mapping approaches described herein can rapidly derive high‐quality THaW mapping products at a landscape extent and resolution high enough to identify, locate and quantify trees, hedgerows, and woodlands in more detail and across larger extents than possible with previous techniques using only spaceborne sensors (Figures 4 and 5), for example (Laurin et al., 2013; Steinhausen et al., 2018).

The baseline THaW data generated showed a high degree of overall positional accuracy (>90%, Kappa 0.93). Although the user's accuracy has a greater range (63% to 98%) when compared to the digitized data, this is likely the result of (1) the THaW Mapping data including canopy gaps that are not distinguished in the manually digitized data and (2) in the very narrow features digitized in the managed hedgerow being more affected by the small parallax distortions evident in the available aerial photography, causing a consistent offset. This latter point is also evidenced by the % agreement included in Table 2, compared to the overall difference between the THaW and digitized areas. These data are also shown to provide sufficient detail to rapidly quantify THaW cover outside of the main woodland areas (Table 2; Figure 6). Figure 4 a and b also highlight the utility of these data to describe the location of “standards” or veteran trees in the landscape (yellow areas) and how these co‐occur with varying hedgerow management, visible as contiguous areas of mapped managed hedgerows (Blue) or larger hedgerows represented as linear woodland analogs (light green). Figure 4c also illustrates separate commercial woodland “compartments” within a continuous woodland area, differentiated by canopy height. Such detail supports those managing the treescape. For example, optimizing tree planting, (Bateman et al., 2022), the enforcement of felling licenses (Stevenson, n.d.), and the expansion of naturally regenerating woodland (Tree, 2017). It may also enable such managers to quantify above and below‐ground C stocks using appropriate existing allometric relationships (Gregg et al., 2021) accepting of course that applying published allometries to generalized treescapes will carry with it some uncertainty linked to species‐specific traits and structural heterogeneity (e.g., hedgerow form can differ from naturalized examples). Given the emerging tree planting agenda for climate change mitigation, such uses also represent an important step in parameterizing and guiding decision making and ensuring that where planting interventions are made, the right trees end up in the right place (Bateman et al., 2022).

There are, however, some important considerations related to the LiDAR dataset used. First, the computation of this baseline requires the gridded LiDAR products appropriate for this context. The Tellus LiDAR used includes a standard DTM product (last return LIDAR pulse classified as ground) and a DSM derived from first return data. These data are used in combination to derive a canopy height model. First return data provide a more robust representation of canopy size as the altimetry measures the topmost canopy structures. However, this terminology is not always consistently applied to LiDAR datasets. For example, data described as a LiDAR DSM is sometimes derived from later or composite returns, as they are optimized for hydrological modeling (Brown et al., 2016). In such cases, the LiDAR DSM data can only effectively describe the densest parts of the canopy geometry (i.e., trunk and inner crown) and may underestimate the THaW canopy extent. However, data are increasingly available that provide first return gridded data, including the UK National Lidar Programme first return DSM data provision (National LIDAR Programme—Data.Gov.Uk, n.d.), which more effectively describes THaW canopy structures.

We have demonstrated that approach presented can be used to map THaW across sub‐county‐level extents very efficiently due to the computational approach used. Despite this efficient workflow, relying solely on episodic LiDAR acquisitions to monitor change in THaW post‐baseline, has limits imposed by data coverage and cost. To address this, we have further demonstrated that baseline THaW products derived from LiDAR data may instead be used to constrain uncertainty in other remote sensing more readily available data and used to track THaW change—for example, satellite SAR. This approach both maximizes the detail of the output mapping, and the temporal dynamics measured.

### 4.2 Aim 2: Monitor canopy change across intra‐ and inter‐annual time steps using freely available Sentinel‐1 SAR data

Here, we demonstrate that the THaW mapping data can be combined with other remote sensing data to provide a spatially explicit mapping of canopy change over short timesteps. The results shown in Figures 7 and 8 demonstrate how canopy change can be measured against a LiDAR‐derived THaW baseline, across large spatial extents without the need for specialist user interpretation. The method illustrated in Figure 2, combines the advantages of very high‐resolution episodic data capture of LiDAR, with the rapid and repeatable and cloud‐independent structural measurements from spaceborne Sentinel‐1 SAR data. This combined approach results in a change detection dataset that has more spatial granularity and shorter temporal intervals, when compared to land use change detection from spaceborne data alone (e.g., Rowland et al., 2020).

To address aim 3, we have also provided a platform to visualize these products within a Google Earth Engine web platform, enabling a more intuitive examination of the outputs without users having to deal with any complex data processing methods (see section 7). Indeed, the methods and tool provided here are already in use by the English Forestry Commission to track and understand whether localized deforestation is within legal allocations (sweep.ac.uk/thaw‐tools/).

When using this tool for the detection of canopy change across multiple timesteps, it is important to consider that it will be sensitive to shifts in landcover and surface moisture (Barrett, 2012; Zhu et al., 2019), which can confound or artificially enhance the detected extents of canopy change. For simplicity, we have attempted to minimize the effect of shifting interannual patterns of moisture and canopy geometry by only comparing matching interannual periods (calendar quarters), when isolating changes in SAR backscatter characteristic of canopy loss. However, when comparator calendar quarters have contrasting average weather conditions across the same extent, the method can still result in high levels of false positive results that manifest as increased “speckle” in the output data. Despite such limitations, with some training to staff within the forestry industry, national parks, and other NGOs, we have enabled non‐technical professionals to understand such patterning and focus on the larger and more contiguous extents of detected change, when interpreting data. At present the tool described here measures canopy loss and changes such as woodland creation occurring outside the baseline THaW area, are omitted. Further work is needed to address this need, particularly the need for a national‐scale product that measures both canopy loss and creation in order to meet the needs of those managing or expanding these treescapes.

### 4.3 Summary and limitations

In summary, this combined approach has important implications for understanding the location, connectivity, and dynamics of existing woody habitats across large landscape areas. While the use of LiDAR provides an excellent opportunity for the detailed mapping shown, the use of S1 SAR data as a proxy for tree cover change over time provides a potential mechanism for monitoring spatial dynamics of THaW cover across landscape extents. Although false positives are a very small fraction of the total mapped features, their inclusion, as discussed in Section 4, is a limitation of the current method that would need addressing in future iterations. The robust removal of permanent buildings using ancillary mapping (open map local–buildings) largely limits the occurrence to smaller features, misrepresented as classes mapped as scrub to hedgerow size. In some areas, larger arable crops (e.g., maize), also result in discontinuous false positive mapping of scrub over field extents, which again represents a limitation of the current method.

Future work will benefit from consideration of other methods of baseline generation, including from SAR data itself, to further improve the responsiveness of such monitoring, and the ability to detect woodland expansion or planting. Currently, the tool cannot detect change due to expansion of THaW, this is also a key limitation that needs to be addressed in future work. Indeed, woodland expansion and land use change are of considerable importance in meeting the objective of net zero by 2050, and in the current political realignment of agricultural policy seen in the UK (Bateman & Balmford, 2018; Olfe‐Kraeutlein et al., 2020).

## 5 CONCLUSION

Herein, we demonstrate that raster‐based workflows applied to airborne lidar data can deliver Tree Hedgerow and Woodland (THaW) baseline mapping across landscape extents rapidly, at 2 m resolution, and with an accuracy of between 91% and 94%. These results highlight that, whilst nearly 90% of the tallest mapped tree canopy (above 15 m) is captured within the National Forest Inventory (NFI), less than half of trees between 3 and 15 m are described in such data. These data may suggest that current estimates of tree distribution are neglecting smaller or less contiguous THaW canopies, which cumulatively account for a significant proportion of the total THaW habitat coverage, landscape carbon stocks, and habitat connectivity. We also demonstrate that using this mapping in combination with Sentinel‐1 SAR data in Google Earth Engine, we can deliver a tool able to track canopy loss rapidly and accurately, at quarterly time intervals selected by the user.

The current toolkit is provided as an open‐access “web app” and can be applied nationally or even internationally, with appropriate THaW baseline data generated as described. This tool and data will allow those managing these landscapes (and their natural capital/ecosystem services) to unlock information on the distribution and loss of these THaW habitats at a temporal and spatial resolution appropriate for their management. In the future, this toolkit could be augmented with a facility to examine THaW expansion. Additionally, it could be expanded across national extents using appropriate LiDAR/SAR data. Such a facility would present a robust and repeatable mechanism of THaW monitoring compatible with future landscape scale management objectives, including the UK ELMS scheme.

## AUTHOR CONTRIBUTIONS

**David J. Luscombe:** Conceptualization (equal); data curation (lead); formal analysis (lead); funding acquisition (supporting); writing – original draft (lead); writing – review and editing (lead). **Naomi Gatis:** Conceptualization (equal); data curation (supporting); formal analysis (supporting); funding acquisition (supporting); writing – original draft (supporting); writing – review and editing (supporting). **Karen Anderson:** Conceptualization (equal); data curation (supporting); formal analysis (supporting); funding acquisition (supporting); writing – original draft (supporting); writing – review and editing (supporting). **Donna Carless:** Conceptualization (equal); data curation (supporting); formal analysis (supporting); funding acquisition (supporting); writing – original draft (supporting); writing – review and editing (supporting). **Richard Brazier:** Conceptualization (equal); data curation (supporting); formal analysis (supporting); funding acquisition (lead); writing – original draft (supporting); writing – review and editing (supporting).

## Data Availability

The accompanying web application displaying both the baseline THaW data and the change detection utility is accessible via: https://davidjluscombe.users.earthengine.app/view/thaw2022luscombe.
